# Therapeutic drug monitoring in clinical practice of early career psychiatrists in Europe: EPA ECPC TDM study results

**DOI:** 10.1192/j.eurpsy.2023.195

**Published:** 2023-07-19

**Authors:** G. Schoretsanitis

**Affiliations:** Department of Psychiatry, Psychotherapy and Psychosomatics, Psychiatric Hospital, University of Zürich, Zurich, Switzerland

## Abstract

**Abstract:**

Therapeutic Drug Monitoring (TDM), i.e. the measurement of blood levels of antipsychotics, is a well-established clinical routine tool in the treatment with antipsychotics including clozapine. Nevertheless, worldwide there are different utilisation cultures and trends with use of TDM varying from regular to very limited. In order to assess attitudes regarding the use and utility of therapeutic drug monitoring in psychiatry trainees and young psychiatrists, the EPA ECPC Task Force on Communication and Publications is performing an online survey consisting of 12 questions previously validated in a british context to gather data on TDM attitudes, practices, and clinical setting.

Apart from capturing early career psychiatrists’ current practices and perspectives regarding antipsychotic TDM the goal of this project is to identify predictive factors for future use of therapeutic drug monitoring (TDM). Here we present the preliminary results of our currently ongoing survey.

**Disclosure of Interest:**

G. Schoretsanitis Consultant of: HLS Therapeutics and ThermoFisher

